# Effect of Nicotinamide Mononucleotide Concentration in Human Milk on Neurodevelopmental Outcome: The Tohoku Medical Megabank Project Birth and Three-Generation Cohort Study

**DOI:** 10.3390/nu16010145

**Published:** 2023-12-31

**Authors:** Yoshie Saito, Keigo Sato, Shinji Jinno, Yoshitaka Nakamura, Takahiro Nobukuni, Soichi Ogishima, Satoshi Mizuno, Seizo Koshiba, Shinichi Kuriyama, Kinuko Ohneda, Masashi Morifuji

**Affiliations:** 1 Wellness Science Labs, Meiji Holdings Co., Ltd., Hachioji 192-0919, Japan; yoshiesaito0520@yahoo.co.jp; 2 Food Microbiology and Function Research Laboratory, Meiji Co., Ltd., Hachioji 192-0919, Japan; keigo.satou@meiji.com (K.S.); shinji.jinno@meiji.com (S.J.); npa@mx4.ttcn.ne.jp (Y.N.); 3 Tohoku Medical Megabank Organization, Tohoku University, Sendai 980-8573, Japan; takahiro.nobukuni@med.tohoku.ac.jp (T.N.); ogishima@megabank.tohoku.ac.jp (S.O.); satoshi.mizuno.e5@tohoku.ac.jp (S.M.); koshiba@megabank.tohoku.ac.jp (S.K.); kuriyama@med.tohoku.ac.jp (S.K.); kohneda@megabank.tohoku.ac.jp (K.O.); 4 Graduate School of Medicine, Tohoku University, Sendai 980-8573, Japan; 5 Advanced Research Center for Innovations in Next-Generation Medicine, Tohoku University, Sendai 980-8573, Japan; 6 International Research Institute of Disaster Science, Tohoku University, Sendai 980-0845, Japan

**Keywords:** nicotinamide mononucleotide, breast milk, infants, neurodevelopmental outcome, cohort study

## Abstract

(1) Background: Breast milk is the only source of nutrition for breastfed infants, but few studies have examined the relationship between breast milk micronutrients and infant neurodevelopmental outcome in exclusively breastfed infants. The aim of this study was to characterize the association between nicotinamide adenine dinucleotide (NAD)-related compounds in the breast milk of Japanese subjects and infant neurodevelopmental outcome. (2) Methods: A total of 150 mother–child pairs were randomly selected from the three-generation cohort of the Tohoku Medical Megabank in Japan. Infants were exclusively breastfed for up to 6 months. Breast milk was collected at 1 month postpartum, and the quantity of NAD-related substances in the breast milk was quantified. The mothers also completed developmental questionnaires at 6, 12, and 24 months. The relationship between the concentration of NAD-related substances in breast milk and developmental indicators was evaluated via ordinal logistic regression analysis. (3) Results: Nicotinamide mononucleotide (NMN) was quantified as the major NAD precursor in breast milk. The median amount of NMN in the breast milk was 9.2 μM. The NMN concentration in breast milk was the only NAD-related substance in breast milk that showed a significant positive correlation with neurodevelopmental outcome in infants at 24 months. (4) Conclusions: The results suggest that NMN in human milk may be an important nutrient for early childhood development.

## 1. Introduction

The so-called first 1000 days, spanning the prenatal period to 2 years after birth, constitute the most significant period of neurodevelopment. Nutritional deficiencies during this period can have long-term and often irreversible effects on neurodevelopmental outcome [1,2]. Nutrients that specifically affect early neurodevelopmental outcome include protein, zinc, iron, choline, folate, iodine, vitamins (A, D, B_6_, and B_12_), and long-chain polyunsaturated fatty acids. Deficiencies in any of these nutrients may have lifelong effects on learning ability, behavior, and emotional regulation [1]. In breastfed infants, breast milk is the sole source of nutrients, but few studies have examined associations between breast milk micronutrients and infant neurodevelopmental outcome in exclusively breastfed children [3].

Nicotinamide dinucleotide (NAD) is an essential pyridine nucleotide that functions as a cofactor and substrate of enzymes that regulates a variety of biological processes, including energy metabolism, gene expression regulation, DNA repair, mitochondrial function, immune function, calcium homeostasis, and cell death [4,5,6]. Decreased NAD levels are associated with cardiovascular and metabolic diseases, neurodegenerative diseases, cancer, and aging [7,8,9]. Thus, the supplementation of NAD precursors to patients who have these specific disease conditions, as well as the elderly, may be beneficial [5,10,11]. Mammalian milk, including human milk, is known to contain high levels of NAD and its precursors [12,13,14,15]. Human milk, in particular, is highly abundant in nicotinamide mononucleotide (NMN) relative to other mammalian species [13]. Meanwhile, studies have reported a rapid increase in the synthesis of NAD and its precursors in the mammary glands of rodents during lactation [16,17].

The roles of the NAD precursors nicotinamide (NAM) and nicotinic acid (NA) have long been studied, but nicotinamide riboside (NR) and NMN have recently received attention as more-effective NAD precursors for raising NAD levels than NA and NAM [18,19]. In rodents, the administration of NR to mothers promotes the neurodevelopment of their offspring [16]. The increased amounts of NAD and its precursors in the mammary glands of NR-treated mothers were associated with enhanced early synaptic pruning and improved physical and neurobehavioral development of the offspring. These effects persisted into adulthood. In rodents, NMN has been reported to improve neurological function in rodent models of age- or disease-related abnormalities, such as Alzheimer’s disease and diabetes [20,21,22,23,24], as well as protect against ischemia-induced brain damage [25,26]. Antidepressant-like activity [27] and neurovascular protection [21,28,29] have also been reported for NMN. However, whether NMN plays a role in normal neurodevelopmental outcome in infants is unclear.

In this study, we measured the quantity of NAD-related substances in the breast milk of Japanese subjects who were randomly selected from a three-generation cohort of the Tohoku Medical Megabank. In addition, the association between the quantity of NAD-related substances in breast milk and infant neurodevelopmental outcome was evaluated using ordinal logistic regression analysis.

## 2. Materials and Methods

### 2.1. Study Design and Population

This study is based on data obtained from the Tohoku Medical Megabank Project Birth and Three-Generation Cohort Study (TMM BirThree Cohort Study), which was a prospective cohort study conducted in Miyagi Prefecture. The design and methods for this study were previously reported by Kuriyama et al. [30]. Between July 2013 and March 2017, 22,493 pregnant women were recruited to participate. Breast milk was collected at 1 month postpartum (median 32 days). The times of milk collection were not controlled. Infants were exclusively breastfed for up to 6 months, and developmental questionnaires were completed at 6, 12, and 24 months. Medical records were obtained for the infants’ length and weight, head circumference, and gestational age.

**Figure 1:**
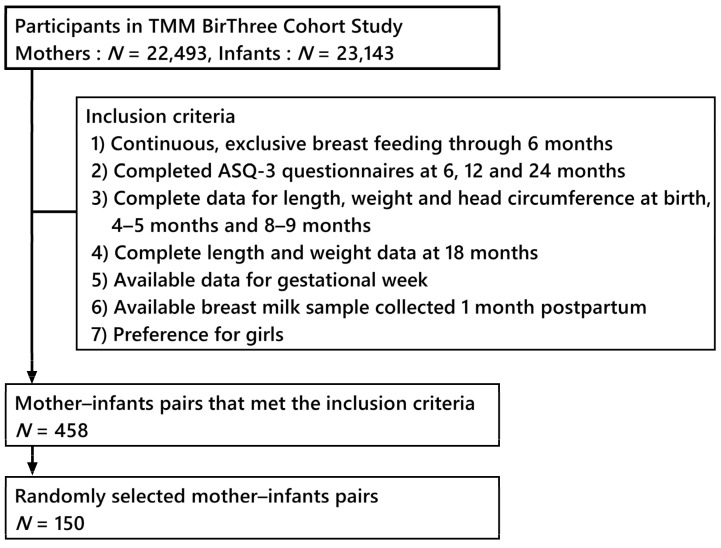
The workflow of selecting mother–infant pairs from the three-generation cohort study.

A workflow concerning the selection of mother–infant pairs from the three-generation cohort study is shown in Figure 1. Mother–infant pairs (*N* = 150) were randomly selected from 458 pairs that met inclusion criteria. The population information is shown in Table 1.

This study was conducted in accordance with the “Ethical guidelines for human genome and gene analysis research” released by the Ministry of Education, Culture, Sports, Science, and Technology (MEXT); the Ministry of Health, Labor, and Welfare (MHLW); and the Ministry of Economy, Trade, and Industry, as well as with the “Ethical guidelines for medical and health research involving human subjects” released by MEXT and MHLW. All procedures involving human participants were approved by the Tohoku University Tohoku Medical Megabank Organization Research Ethics Review Board (approval number 2021-4-101). The use of breast milk and detailed information deposited in the integrated biobank was approved by the sample and data access committee of the Tohoku Medical Megabank Project. Before beginning the study, detailed information was disclosed on the Tohoku Medical Megabank Organization website to provide the patients an opportunity to refuse to participate.

### 2.2. Measurement of NAD-Related Compounds Using HPLC-MS/MS

Breast milk samples collected at 1 month postpartum (mature milk) were obtained from the TMM integrated biobank. The breast milk used in this study was maintained at 4 °C for up to 15 h from the time it was sampled at the medical facility until it was stored at −80 °C at the research facility [31].

NAD-related compounds in breast milk were analyzed using modified versions of methods from previous reports [12,14,32]. Human milk samples were thawed for 10 min in a 40 °C water bath with sonication applied. A 40 μL aliquot was mixed with 280 μL of internal standard solution and 1280 μL of methanol. The mixture was vortexed for 1 min and centrifuged for 10 min at 4 °C at 17,900× *g*. A 50 μL aliquot of supernatant was evaporated using a miVac concentrator (Genevac, Ipswich, United Kingdom) at 37 °C. The dried extract was mixed with 525 μL of 0.1 mg/mL of citric acid, allowed to stand for 15 min on ice, and reconstituted via vortexing for 2 min. Then, 500 μL of chloroform was added to the reconstituted extract, which was vortexed for 2 min, allowed to stand for 15 min on ice, and centrifuged at 10,500× *g* for 10 min at 4 °C. The supernatant was filtered through a 0.2 μm filter and analyzed using high-performance liquid chromatography–tandem mass spectrometry (HPLC-MS/MS, HPLC; ACQUITY H-class Bio Binary system, Waters, MS/MS; Xevo TQ-XS, Waters Corporation, Milford, MA, USA).

All analyses were performed on a 2.1 × 150 mm column with a 1.8 µm particle size (ACQUITY UPLC Premier HSS T3, Waters Corporation). Mobile phases A and B consisted of 5 mM each of ammonium formate and acetonitrile, respectively. The initial eluent composition was 100% A, which increased to 10% B over 3 min. The 10% B concentration was maintained for 0.5 min and then increased to 90% B over 1 min, held at 90% B for 3.5 min, and then reduced to 0% B over 3 min. The total run time was 11 min. The eluent flow was 0.3 mL/min, and the column was maintained at 45 °C. Analytes were detected using electrospray ionization in positive mode.

### 2.3. Method Validation

Matrix-matched calibration was applied for each analyte. Calibration curves were constructed after the subtraction of endogenous content. Outliers were marked and excluded from the calibration process when an individual point deviated by >20% (25% for the lowest calibration point) from the theoretical value.

To compare the efficacy of an existing sample preparation, analyte recovery and ion suppression values were determined by comparing peak areas for three types of samples: (1) human milk spiked before sample preparation with a known amount of analyte (pre-extraction) after the deduction of the endogenous analyte content; (2) human milk spiked after sample preparation with a known amount of analyte (post-extraction) after the deduction of the endogenous analyte content; and (3) a standard solution of pure analytes. A peak area ratio of 2 to 3 between samples reflects the extent of ion suppression. A peak area ratio of 1 to 2 between samples reflects recovery. Recovery studies were carried out by individually preparing and quantifying a QC sample 3 times (*N* = 3) within one batch at both levels. The average of the results within the calibrated range in human milk was reported.

To evaluate the performance attainable using this method, inter-day precision of the QC samples was expressed as a coefficient of variation (%). Inter-day repeatability studies were carried out by individually preparing and quantifying QC samples in duplicate on four consecutive days (*N* = 4).

### 2.4. Stability Test

To evaluate the stability of NAD-related compounds in human milk, pooled human milk (SRM1953, NIST) spiked with NMN, NR, and NA was kept at 4 °C for 0 to 14 days before being frozen at −80 °C. All frozen samples were analyzed on the same day.

### 2.5. Developmental Outcomes of Infants

Infant developmental outcomes were assessed using the Ages and Stages Questionnaire 3 (ASQ-3), which was administered to the parents when the children were 6, 12, and 24 months old. The ASQ-3 is used to screen infants aged 1–66 months for risk of developmental delay in five developmental domains: communication, gross motor skills, fine motor skills, problem-solving ability, and personal and social skills [33,34]. For each domain, six questions are used to assess achievements in the associated skills. The responses “yes”, “sometimes”, and “not yet” correspond to scores of 10, 5, and 0, respectively. The total score thus ranges from 0 to 60 for the five domains. The ASQ-3 scores were categorized into 13 levels (0, 5, 10, 15, 20, 25, 30, 35, 45, 50, 55, and 60) because the scores consisted of values of 0–60, in increments of 5 points. The questions used to determine the five developmental domains vary with the age of the child in months.

### 2.6. Physical Growth Outcomes of Infants

The physical growth of the infants at 1, 5, 9, and 18 months of age was obtained from medical records. Each month encompassed between 15 and 44 days, 120 and 179 days, 240 and 299 days, and 540 and 599 days, respectively. When multiple data on physical growth were available within each evaluation month, the data from the earliest consultation date were used.

### 2.7. Confounders

Covariates were chosen a priori based on previous research. The paper referenced is listed in the ASQ-3 guidebook as a paper reporting a confounding factor affecting the ASQ-3 [35]. The report referenced is a meta-analysis showing confounding factors associated with breast milk and child development [36].

Factors considered potential confounders of the ASQ-3 results were included: gestational week, household income, alcohol consumption during pregnancy, and number of children. Variables related to the gestational weeks of the infants were obtained from medical records. Annual household income (<JPY 2,000,000; JPY 2,000,000–3,999,999; 4,000,000–5,999,999; 6,000,000–7,999,999; 8,000,000–9,999,999; 10,000,000–11,999,999; and ≥12,000,000) was obtained as ordinal variables from the second-trimester questionnaire (1–7). Variables related to alcohol consumption during pregnancy were obtained from the first- and second-trimester questionnaires as two variables: presence or absence of alcohol consumption during pregnancy. Variables related to the number of children in a family were obtained as ordinal variables from the enrolment questionnaire.

Factors considered potential confounders for infant physical growth were age in days, gestational week, household income, alcohol consumption during pregnancy, and number of children. The infant’s age in days on the day of examination was calculated by subtracting the infant’s date of birth from the chart.

### 2.8. Statistical Analysis

The correlation between concentrations of NAD-related compounds was analyzed using Spearman’s correlation test. The association between concentrations of NAD-related compounds in breast milk and infants’ developmental outcomes was evaluated using Spearman’s rank correlation as a crude analysis. Furthermore, the association was evaluated using an ordinal logistic regression analysis to calculate the common odds ratio and 95% CI. Evaluations were performed using a crude (unadjusted) model and an adjusted model. Three NAD metabolites and five ASQ-3 questionnaire items were corrected for multiplicity, using the Benjamini–Hochberg method (FDR). NA and NR were excluded from the correlation analysis because many values were below the detection limit.

The associations between concentrations of NMN in breast milk and repeated measures of infants’ growth outcomes were evaluated using linear mixed-effects models with random effects for subjects to calculate the coefficient and 95% confidence interval (CI). Infants whose physical growth data did not correspond to the definition of age in months for the evaluation (1 month, 15–44 days; 5 months, 120–179 days; 9 months, 240–299 days; and 18 months, 540–599 days) were excluded from each statistical analysis.

Mother–infant pairs with incomplete background information were excluded from each statistical analysis. Breast milk in which no NAD-related compounds were detected was treated as 0 μM. In all analyses, *p* < 0.05 was considered statistically significant. All statistical analyses were carried out using R statistical software (version 4.2.2).

Additional packages were used for calculating the Z-scores of the anthropometric indices of the infants (zscorer), ordinal logistic regression analysis (MASS), and linear mixed-effects models (lme4).

## 3. Results

The mass spectrometric behavior of all standards and internal standards was studied using positive-ion ESI. Compound-dependent parameters are summarized in Supplementary Table S1. The matrix-matched calibration range, recovery, ion suppression, and precision results are summarized in Supplementary Table S2. NAMN could not be calibrated due to differences in ion suppression between the selected internal standards. All NAD metabolites exhibited recovery ranging between 90 and 105% and an inter-day precision ranging between 1.0 and 12.2%. These results indicated that the extraction procedure used for this study is very well suited for these compounds. Ion enhancement neared 56% for NMN and did not affect the targeted quantification range. LC-MS/MS chromatograms of the standard and human milk samples are shown in Supplementary Figure S1A.

In the TMM BirThree Cohort Study, breast milk samples were stored at 4 °C from the time of collection until freezing at −80 °C. Since the storage period at 4 °C could vary between samples, the stability of NAD-related substances in breast milk at 4 °C was confirmed in order to provide a suitable consideration of the concentration of NAD-related substances in breast milk. The concentrations of NAD-related substances in breast milk samples stored at 4 °C for 0, 1, 2, 3, 7, 10, and 14 days are shown in Supplementary Figure S1B. The concentrations of NMN and NAD in the breast milk did not change during the storage period, whereas the NAD concentration decreased slightly. Meanwhile, the NR concentration in the breast milk decreased significantly, and NAM increased, but the total concentration of NAM and NR remained unchanged, suggesting that NR may have been degraded to NAM.

Peaks for NMN, NAD, NAM, and NR were detected in all samples. NA, methylnicotinamide (MeNAM), and nicotinic acid mononucleotide (NAMN) were undetectable (denoted as 0 in the figure) in most of the samples. Nicotinamide adenine dinucleotide phosphate (NADP) and nicotinic acid adenine dinucleotide (NAAD) were not detectable using our assay method; thus, no peaks were detected in any of the samples. For NMN, NAD, NAM, NR, and NA, for which peaks were detected within the calibrated concentration range, the concentrations were comparable at the order level to values reported in previous studies [12,13,15]. The values for MeNAM were outside of the calibrated concentration range. The NAMN concentration could not be calculated, as this compound was not calibrated.

**Figure 2:**
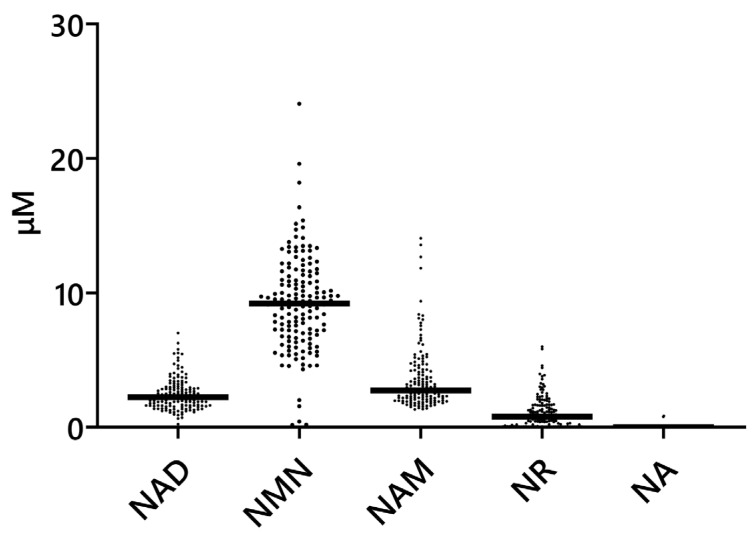
Concentrations of NAD-related compounds in breast milk. Each point represents an individual sample (*N* = 150), and the horizontal bar indicates the median value (NAD, 2.2 μM; NMN, 9.2 μM; NAM, 2.7 μM; NR, 0.8 μM; and NA, 0 μM). NAD: nicotinamide adenine dinucleotide. NMN: nicotinamide mononucleotide. NAM: nicotinamide. NR: nicotinamide riboside. NA: nicotinic acid.

The concentrations of NMN, NAD, NAM, NA, and NR in the breast milk samples are shown in Figure 2. Quantiles of NAD-related substances in breast milk are shown in Supplementary Table S3. NMN was the most abundant NAD-related substance in breast milk, and the concentrations of some NAD-related substances in the breast milk were significantly correlated with each other (Supplementary Figure S2). The NAD and NMN, as well as NAD and NAM concentrations, showed significant positive correlations.

Next, we examined the ASQ-3 results at 6 months, 12 months, and 24 months for the infants in this study (Table 2). Spearman’s rank correlation coefficients describing the relationship between the concentration of NAD-related substances in the breast milk samples and developmental indicators showed a significant positive correlation between NMN and all developmental indicators at 24 months (Table 3). Therefore, we evaluated the association between NAD-related substance content in breast milk and developmental indicators, using ordinal logistic regression. In both the crude model and adjusted model, there was a significant positive correlation between the NMN concentration in breast milk and developmental indicators at 24 months (Table 4). Similar results were obtained in the population, excluding infants born at low birth weight (<2500 g) and with a gestational week of less than 37 weeks (Supplementary Tables S4 and S5).

On the other hand, there were significant negative correlations between NAM and some developmental indicators at 6 and 24 months, as assessed using Spearman’s rank correlation coefficient (Table 3).

Table 5 shows the results of the physical development scores for 0-, 1-, 5-, 9-, and 18-month-old infants in this study. The results yielded by the linear mixed-effects models evaluating the relationship between the NMN concentration in breast milk and repeated measures of developmental indices are shown in Table 6. No significant correlations were found between the NMN concentration in breast milk and growth indices with an adjustment for both the crude model by age in days and the adjusted model by age in days, gestational week, household income, alcohol consumption during pregnancy, and number of children.

## 4. Discussion

In this study, a positive correlation was observed between NMN concentration in human milk and neurodevelopmental outcome in children, indicating that NMN may be an important nutrient for human neurodevelopmental outcome. 

Several reports have indicated that NAM or niacin is the main NAD precursor in breast milk [12,13,14], but statistical comparisons are difficult since these earlier studies had small sample sizes (*N* = 4~8), and the micronutrient concentrations in the breast milk varied with maternal nutrition and health status [37,38,39]. This study is, to the best of our knowledge, the first to examine the concentration of NAD-related substances in breast milk on a large scale. Here, we showed that NMN is the major NAD precursor in breast milk. Individual differences in NMN concentration could be particularly large. The breast milk used in this study was maintained at 4 °C for up to 15 h between sampling at the medical facility and storage at −80 °C at the research facility [31]. The NMN concentrations were likely maintained since NMN generally exhibits good stability and is stable in plasma, cell culture media, and milk [13,18,40,41]. Here, we showed that NMN was indeed stable in breast milk at 4 °C for at least 2 weeks, suggesting that the variation in NMN concentrations we observed in this study is not due to differences in breast milk storage conditions but rather reflects variations in the original NMN concentration in breast milk. On the other hand, the amount of NR was substantially reduced, while the NAM concentration increased in breast milk stored at 4 °C. The sum of the NR and NAM concentration remained constant, suggesting that NR decomposed into NAM. This possibility is consistent with large increases in NAM observed in mice that either orally ingested NR or that received intraperitoneal NR, as well as via culturing in isolated mouse plasma [18,19]. In contrast to the kinetics in human milk, NR is stable, and it is the major NAD metabolite in cow milk [42,43], suggesting that factors that degrade NR may be absent in cow milk but not in human milk.

Three mammalian NAD+ biosynthetic metabolic pathways are known. The first is a de novo pathway that begins with L-tryptophan. The second is a pris-handler pathway starting with NA, and the third is a salvage pathway that regenerates consumed NAD+. In mammalian cells, the salvage pathway produces the majority of NAD+. In this salvage pathway, the rate-limiting enzyme nicotinamide phosphoribosyltransferase (NAMPT) converts NAM into NMN. NMN can also be synthesized from NR by NR kinase (NRK). NMN is converted to NAD+ by NMN adenylyltransferase (NMNAT) [44]. In the present study, the concentrations of NAM and NMN in human milk were negatively correlated, presumably due to differences in the activity of NAMPT, the rate-limiting step in the salvage pathway.

It may be reasonable to assume that NMN is the major NAD precursor in breast milk. The first NAD precursors, NA and NAM, have been used clinically for many years to treat pellagra and are also available on the world market as food supplements, but both produce side effects at high dosages [5,45]. NA has been reported to cause flushing, headache, dizziness, gastrointestinal distress, and general systemic vasodilation, whereas NAM has been reported to cause nausea and vomiting, as well as several other side effects. NAM is also a feedback inhibitor of NAD+-dependent enzymes, and high concentrations of NAM inhibit the activity of poly (ADP-ribose) polymerase (PARP), sirtuins, and CD38 [46], resulting in an increase in accumulation of hepatic fat [47,48], insulin resistance [49], and spatial-learning deficits [50]. On the other hand, no adverse effects were observed regarding NMN and NR even after long-term ingestion of high doses [51,52]. Furthermore, NR and NMN are more effective than NA and NAM at increasing tissue NAD levels when administered orally or intraperitoneally [18,19]. Thus, NR and NAM are effective NAD boosters that have few side effects. Since NMN is more stable than NR in breast milk, NMN may be a suitable source of NAD precursors in breast milk. 

NMN has been reported to have a number of beneficial effects on neurological function, but all of these effects have been demonstrated in the elderly or under disease conditions. The supplementation of NR to the mother has been shown to be effective in increasing infant neurodevelopment, but increases in NMN levels related to NR supplementation were confirmed in the mammary glands and not in breast milk [16]. In the present study, we demonstrated, for the first time, an association between NMN levels in breast milk and infant neurodevelopmental outcome. There are multiple possible mechanisms by which NMN may be related to neurodevelopmental outcome. Important processes in human neurodevelopment that occur during the first two years of life include neuronal proliferation, axon and dendrite growth, synapse formation, and myelination [44,45], and all of these processes can involve NAD synthase or NAD+-dependent enzymes. For instance, NAMPT is associated with neural stem cell proliferation [53]; PARP1 is associated with neuronal proliferation, migration, and adhesion [54]; NMNAT is associated with dendrite and axon elongation and branching [55,56,57,58]; SIRT2 is associated with neuronal migration and dendritic development and myelination [59,60,61]; and SIRT6 is associated with dendrite development [62]. NMN in human milk could affect infant development through these processes. ASQ-3 endpoints also include motor function, but NAD is involved in neuromuscular junction and muscle structure and function, as well [63,64,65]. Thus, NMN in breast milk may improve child development not only through its effects on the central nervous system but also on the peripheral nervous system and muscles.

In this study, there was no correlation between ASQ-3 and NMN at 6 and 12 months of age, and only ASQ-3 at 24 months of age correlated with NMN. In a study examining structural and cognitive differences in the neurodevelopmental outcome of infants that had different nutritional intakes, differences began to emerge at around 18 months of age [66]. Another study found that human milk oligosaccharide concentrations in breast milk given to 1-month-old infants were associated with cognitive development at 24 months [67]. These findings suggest that the effect of the NMN concentration in breast milk is not apparent at 6 and 12 months of age. We also observed significant negative correlations between NAM and several developmental measures at 6 and 24 months that may be related to the negative feedback activity of NAM, as described above.

There are several limitations of this observational study. First, because this is an observational study, the causal relationship between factors and outcomes is unclear. Second, this study focused on girls. Gender differences have been reported in terms of the structure and neurodevelopmental outcome during infancy and the effects of nutrition on neurodevelopmental outcome [16,68,69,70], so additional studies are needed to determine whether a similar association is present in boys. Third, the ASQ-3 was primarily developed to screen for developmental delay rather than to serve as a benchmark for normal development. However, the ASQ-3 has been standardized around the world and used for various purposes in different studies. Fourth, the absolute value of NAD-related substance intake is unknown since the concentration of NAD-related substances was evaluated only in the first month of lactation and the intake of breast milk by the infants is unknown. The 1-month lactation period does fall within the normal milk period, and breast milk produced during this period has a stable nutritional composition and thus should be a suitable representative breast milk sample. In addition, because the study included only infants who were exclusively breastfed during the first 6 months of life, large differences in the amount of breast milk consumed were minimized, and the relative intake of NAD-related substances could be fully evaluated. Fifth, we focused only on NAD-related substances in breast milk components, but the possibility that other milk components also had an effect on the results cannot be excluded. Sixth, although milk samples and data for a large number of parent–infant pairs (n = 22,492) were kept in this cohort and 458 pairs satisfied the inclusion criteria, only a subset of the samples (n = 150) could be analyzed due to limited research funding. However, Tohoku Medical Megabank is Japan’s only biobank, unique in the world, that possesses breast milk samples and data on growth and development outcome. Although the sample size was small, these are valuable data that show, for the first time, a relationship between NMN in milk and neurodevelopmental outcomes.

## 5. Conclusions

In conclusion, in this study, the concentration and distribution of NAD-related substances in human milk were further described. Consistent with previous findings, NMN had the highest concentrations in breast milk among the NAD-related substances. We demonstrated, for the first time, a positive relationship between NMN concentrations in breast milk and the neurodevelopmental outcome of a child. NMN is known to exhibit anti-aging effects, and these results suggest that this compound may also have important roles even in infancy. Further studies are needed to clarify why the levels of NMN breast milk are high and the mechanism whereby NMN in breast milk affects infant development.

## 6. Patents

The authors have applied for a patent corresponding to the contents of this paper.

## Figures and Tables

**Table 1:** Characteristics of the mother–infant pairs.

	*N*	Mean ± SD
		or %
Mother	150	
Age during participation, years	150	31.6 ± 4.5
Body mass index, kg/m^2^	146	20.6 ± 2.9
No information	4	2.70%
Household income, JPY/year		
<2,000,000	2	1.3%
2,000,000–3,999,999	38	25.3%
4,000,000–5,999,999	47	31.3%
6,000,000–7,999,999	32	21.3%
8,000,000–9,999,999	18	12.0%
10,000,000–11,999,999	6	4.0%
≥12,000,000	3	2.0%
No information	4	2.7%
Employment status (no), %		
At 6 months	95	63.30%
No information	7	4.70%
At 12 months	71	47.30%
No information	15	10.00%
At 24 months	66	44.00%
No information	5	3.30%
Alcohol consumption during pregnancy (yes), %	32	21.30%
No information	3	2.00%
Number of children in the family (people)		
1	71	47.3%
2	58	38.7%
3	20	13.3%
4	1	0.7%
No information	0	0%
Infants	150	
Sex (female), %	150	100%
At birth		
Gestational weeks, week	150	39.3 ± 1.2
<37	2	1.30%
≥42	0	0%
Weight, g	150	3006.0 ± 372.8
<2500	16	10.70%
≥4000	1	0.70%
Length, cm	150	49.0 ± 2.1
Head circumference, cm	150	33.4 ± 1.4
At 1 month (age in 15–44 days)		
Age, days	124	31.6 ± 3.6
Weight, kg	124	4.0 ± 0.5
Length, cm	124	52.9 ± 2.2
Head circumference, cm	121	36.5 ± 1.2
Chest circumference, cm	82	35.6 ± 1.7
Out of age in days	26	17.30%
At 5 months (encompassing 120–179 days)		
Age, days	145	144.5 ± 15.6
Weight, kg	145	6.8 ± 0.7
Length, cm	142	63.2 ± 2.4
Head circumference, cm	139	41.3 ± 1.6
Chest circumference, cm	137	41.4 ± 1.7
Out of age in days	5	3.30%
At 9 months (encompassing 240–299 days)		
Age, days	138	270.0 ± 15.7
Weight, kg	138	8.1 ± 0.8
Length, cm	138	68.6 ± 2.2
Head circumference, cm	135	43.8 ± 1.5
Chest circumference, cm	123	43.8 ± 2.0
Out of age in days	12	8.00%
At 18 months (encompassing 540–599 days)		
Age, days	134	563.0 ± 11.7
Weight, kg	134	9.9 ± 1.0
Length, cm	134	78.2 ± 6.6
Out of age in days	16	10.70%

**Table 2:** Results of Ages and Stages Questionnaire 3 (ASQ-3).

	6 Month		12 Month		24 Month	
ASQ-3 Score	*N*	Median (IQR)	*N*	Median (IQR)	*N*	Median (IQR)
Communication	150	50 (40, 55)	150	45 (35, 53.8)	150	60 (50, 60)
Gross motor	150	40 (30, 50)	150	52.5 (35, 60)	150	60 (51.3, 60)
Fine motor	150	50 (35, 60)	150	50 (45, 60)	150	50 (50, 60)
Problem solving	150	55 (50, 60)	150	50 (40, 60)	150	55 (50, 60)
Personal–social	150	45 (30, 55)	150	40 (35, 55)	150	50 (45, 50)

Scores were expressed as a range from 0 to 60, in increments of 5 points. Higher scores indicate more positive outcomes. IQR: Interquartile range.

**Table 3:** Relationship between concentration of NAD-related substances in breast milk and developmental indicators, as evaluated using Spearman’s rank correlation coefficient.

ASQ-3	NAD		NMN		NAM	
6 months						
Communication	r = 0.107, Q = 0.486		r = 0.016, Q = 0.910		r = 0.060, Q = 0.756	
Gross motor	r = −0.035, Q = 0.773		r = −0.055, Q = 0.756		r = −0.232, Q = 0.032	*
Fine motor	r = −0.086, Q = 0.612		r = −0.035, Q = 0.773		r = −0.182, Q = 0.128	
Problem solving	r = −0.108, Q = 0.486		r = 0.002, Q = 0.985		r = −0.234, Q = 0.032	*
Personal–social	r = −0.081, Q = 0.612		r = −0.041, Q = 0.773		r = −0.135, Q = 0.372	
12 months						
Communication	r = 0.069, Q = 0.699		r = 0.108, Q = 0.699		r = −0.048, Q = 0.699	
Gross motor	r = 0.065, Q = 0.699		r = 0.077, Q = 0.699		r = −0.130, Q = 0.699	
Fine motor	r = 0.054, Q = 0.699		r = 0.056, Q = 0.699		r = −0.052, Q = 0.699	
Problem solving	r = −0.011, Q = 0.957		r = 0.075, Q = 0.699		r = −0.122, Q = 0.699	
Personal–social	r = −0.023, Q = 0.898		r = −0.004, Q = 0.957		r = −0.121, Q = 0.699	
24 months						
Communication	r = 0.198, Q = 0.038	*	r = 0.199, Q = 0.038	*	r = 0.016, Q = 0.907	
Gross motor	r = 0.077, Q = 0.527		r = 0.186, Q = 0.048	*	r = −0.126, Q = 0.234	
Fine motor	r = 0.025, Q = 0.880		r = 0.208, Q = 0.038	*	r = −0.002, Q = 0.981	
Problem solving	r = 0.056, Q = 0.675		r = 0.288, Q = 0.005	*	r = −0.077, Q = 0.527	
Personal–social	r = 0.048, Q = 0.699		r = 0.259, Q = 0.010	*	r = −0.211, Q = 0.038	*

* Q < 0.05. NAD: nicotinamide adenine dinucleotide. NMN: nicotinamide mononucleotide. NAM: nicotinamide. ASQ: Ages and Stages Questionnaire.

**Table 4:** Association between NAD-related substances in breast milk and developmental indicators determined using ordinal logistic regression.

6-Month ASQ-3	Crude			Adjusted		
	cOR (95% CI)	Q Value		cOR (95% CI)	Q Value	
NAD						
Communication	1.15 (0.90, 1.46)	0.772		1.20 (0.93, 1.55)	0.812	
Gross motor	0.91 (0.71, 1.17)	0.886		0.95 (0.72, 1.24)	0.815	
Fine motor	0.89 (0.69, 1.15)	0.772		1.00 (0.76, 1.32)	0.999	
Problem solving	0.88 (0.68, 1.14)	0.772		0.93 (0.70, 1.25)	0.815	
Personal–social	0.87 (0.68, 1.11)	0.772		0.89 (0.68, 1.16)	0.815	
NMN						
Communication	1.00 (0.92, 1.08)	0.930		0.99 (0.91, 1.08)	0.956	
Gross motor	0.98 (0.91, 1.06)	0.930		0.98 (0.90, 1.06)	0.815	
Fine motor	0.99 (0.91, 1.08)	0.930		0.97 (0.89, 1.06)	0.815	
Problem solving	0.99 (0.92, 1.08)	0.930		0.98 (0.90, 1.07)	0.815	
Personal–social	0.99 (0.91, 1.07)	0.930		0.98 (0.89, 1.07)	0.815	
NAM						
Communication	1.01 (0.89, 1.14)	0.930		0.97 (0.84, 1.13)	0.815	
Gross motor	0.86 (0.75, 0.98)	0.216		0.84 (0.71, 0.98)	0.366	
Fine motor	0.87 (0.77, 0.99)	0.216		0.87 (0.75, 1.00)	0.430	
Problem solving	0.96 (0.84, 1.09)	0.899		0.95 (0.81, 1.11)	0.815	
Personal–social	0.93 (0.83, 1.04)	0.772		0.97 (0.84, 1.13)	0.815	
12-month ASQ-3	Crude			Adjusted		
	cOR (95% CI)	Q value		cOR (95% CI)	Q value	
NAD						
Communication	1.18 (0.93, 1.51)	0.909		1.22 (0.94, 1.58)	0.730	
Gross motor	1.12 (0.87, 1.45)	0.909		1.23 (0.94, 1.61)	0.730	
Fine motor	1.06 (0.83, 1.36)	0.909		1.02 (0.78, 1.34)	0.937	
Problem solving	0.97 (0.76, 1.23)	0.909		1.01 (0.78, 1.31)	0.937	
Personal–social	0.99 (0.78, 1.25)	0.909		0.99 (0.77, 1.27)	0.937	
NMN						
Communication	1.05 (0.97, 1.14)	0.909		1.07 (0.98, 1.17)	0.730	
Gross motor	1.05 (0.96, 1.14)	0.909		1.06 (0.97, 1.16)	0.785	
Fine motor	1.03 (0.95, 1.12)	0.909		1.01 (0.92, 1.10)	0.937	
Problem solving	1.02 (0.94, 1.11)	0.909		1.01 (0.92, 1.11)	0.937	
Personal–social	0.99 (0.92, 1.07)	0.909		0.99 (0.91, 1.08)	0.937	
NAM						
Communication	1.01 (0.90, 1.14)	0.909		0.96 (0.83, 1.12)	0.937	
Gross motor	0.93 (0.82, 1.05)	0.909		0.95 (0.81, 1.11)	0.937	
Fine motor	0.98 (0.87, 1.11)	0.909		0.96 (0.83, 1.11)	0.937	
Problem solving	0.94 (0.84, 1.06)	0.909		0.95 (0.82, 1.10)	0.937	
Personal–social	1.01 (0.89, 1.14)	0.909		0.98 (0.85, 1.13)	0.937	
24-month ASQ-3	Crude			Adjusted		
	cOR (95% CI)	Q value		cOR (95% CI)	Q value	
NAD						
Communication	1.35 (1.02, 1.79)	0.077		1.28 (0.95, 1.71)	0.170	
Gross motor	1.28 (0.96, 1.72)	0.168		1.40 (1.02, 1.93)	0.088	
Fine motor	1.08 (0.85, 1.39)	0.596		1.13 (0.86, 1.48)	0.474	
Problem solving	1.14 (0.87, 1.48)	0.486		1.25 (0.94, 1.67)	0.191	
Personal–social	1.13 (0.86, 1.47)	0.486		1.28 (0.95, 1.72)	0.170	
NMN						
Communication	1.13 (1.03, 1.24)	0.034	*	1.15 (1.04, 1.28)	0.024	*
Gross motor	1.13 (1.03, 1.24)	0.034	*	1.16 (1.04, 1.29)	0.024	*
Fine motor	1.12 (1.03, 1.22)	0.034	*	1.14 (1.04, 1.24)	0.024	*
Problem solving	1.20 (1.09, 1.31)	0.003	*	1.18 (1.07, 1.30)	0.008	*
Personal–social	1.15 (1.05, 1.26)	0.015	*	1.21 (1.09, 1.34)	0.004	*
NAM						
Communication	1.04 (0.91, 1.19)	0.596		1.00 (0.86, 1.17)	0.952	
Gross motor	0.93 (0.81, 1.06)	0.477		0.92 (0.78, 1.08)	0.404	
Fine motor	1.02 (0.90, 1.15)	0.799		1.01 (0.87, 1.16)	0.952	
Problem solving	0.95 (0.84, 1.07)	0.486		0.96 (0.83, 1.10)	0.629	
Personal–social	0.87 (0.77, 0.99)	0.077		0.85 (0.72, 0.99)	0.092	

Crude model: (*N* = 150). Adjusted model: adjusted for gestational week, household income, alcohol consumption during pregnancy, and number of children (*N* = 144). * Q < 0.05. NAD: nicotinamide adenine dinucleotide. NMN: nicotinamide mononucleotide. NAM: nicotinamide. ASQ: Ages and Stages Questionnaire.

**Table 5:** Physical development scores for 0-, 1-, 5-, 9-, and 18-month-old infants.

	*N*	Median (IQR)	Mean ± SD
At birth			
Weight-for-length Z-score	143	−0.5 (−1.3, 0.1)	−0.7 ± 1.2
Length-for-age Z-score	150	−0.1 (−0.7, 0.7)	−0.1 ± 1.1
Weight-for-age Z-score	150	−0.4 (−1.2, 0.1)	−0.5 ± 0.9
Head-circumference-for-age Z-score	150	−0.3 (−1.2, 0.1)	−0.4 ± 1.2
1 months			
Weight-for-length Z-score	124	0.0 (−0.6, 0.7)	0.0 ± 1.0
Length-for-age Z-score	124	−0.5 (−1.2, 0.3)	−0.5 ± 1.1
Weight-for-age Z-score	124	−0.4 (−1.1, 0.3)	−0.4 ± 0.9
Head-circumference-for-age Z-score	121	−0.1 (−0.6, 0.7)	−0.1 ± 1.0
5 months			
Weight-for-length Z-score	142	0.2 (−0.2, 0.8)	0.2 ± 0.9
Length-for-age Z-score	142	0.0 (−0.7, 0.6)	−0.2 ± 1.0
Weight-for-age Z-score	145	0.2 (−0.6, 0.6)	0.0 ± 0.9
Head-circumference-for-age Z-score	139	0.0 (−0.7, 0.8)	0.0 ± 1.2
9 months			
Weight-for-length Z-score	138	0.2 (−0.4, 0.8)	0.2 ± 0.9
Length-for-age Z-score	138	−0.6 (−1.1, 0.1)	−0.6 ± 0.9
Weight-for-age Z-score	138	−0.1 (−0.9, 0.5)	−0.2 ± 0.9
Head-circumference-for-age Z-score	135	0.1 (−0.5, 0.7)	0.1 ± 1.1
18 months			
Weight-for-length Z-score	133	0.0 (−0.6, 0.6)	0.1 ± 0.9
Length-for-age Z-score	134	−0.8 (−1.4, −0.3)	−1.0 ± 2.3
Weight-for-age Z-score	134	−0.4 (−0.9, 0.1)	−0.4 ± 0.8

**Table 6:** Linear mixed-effects models evaluating the relationship between nicotinamide mononucleotide (NMN) concentration in breast milk and repeated measures of developmental indices.

	Crude		Adjusted	
	β (95% CI)	*p*-Value	β (95% CI)	*p*-Value
NMN				
Weight-for-length Z-score	−0.005 (−0.034, 0.025)	0.763	−0.001 (−0.033, 0.030)	0.931
Length-for-age Z-score	−0.018 (−0.063, 0.027)	0.428	−0.032 (−0.075, 0.010)	0.136
Weight-for-age Z-score	−0.018 (−0.050, 0.014)	0.262	−0.029 (−0.059, 0.002)	0.066
Head-circumference-for-age Z-score	−0.027 (−0.068, 0.014)	0.191	−0.037 (−0.077, 0.002)	0.060

Crude model: adjusted for age in days as fixed effects and for subject ID as a random effect. Adjusted model: adjusted for age in days, gestational week, household income, alcohol consumption during pregnancy, and number of children as fixed effects and for subject ID as a random effect.

## Data Availability

The data presented in this study are available upon request from the corresponding author. The data are not publicly available since they contain confidential information.

## Supplementary Materials

The following supporting information can be downloaded at https://www.mdpi.com/article/10.3390/nu16010145/s1. Supplementary Table S1: Parameters for HPLC–MS/MS analysis of NAD-related compounds; Supplementary Table S2: Matrix-matched calibration range, recovery, ion suppression, and precision of NAD-related compounds; Supplementary Table S3: Quantiles of NAD-related substances in breast milk; Supplementary Table S4: Association between NAD-related substances in breast milk and developmental indicators determined via ordinal logistic regression. Analysis of mother–infant pairs excluding infants with a gestational age of less than 37 weeks; Supplementary Table S5: Association between NAD-related substances in breast milk and developmental indicators determined using ordinal logistic regression. Analysis of mother–infant pairs excluding infants with low birth weight (<2500 g); Supplementary Figure S1: (A) Analysis of NAD-related substances in breast milk samples, (B) Measurement of the stability of NAD-related substances; Supplementary Figure S2: Correlation of NAD-related compounds in human milk (Speaman’s correlation test).
