# Supplementary material for: Effect of Nicotinamide Mononucleotide Concentration in Human Milk on Neurodevelopmental Outcome: The Tohoku Medical Megabank Project Birth and Three-Generation Cohort Study
Source: Nutrients. 2023 Dec 31;16(1):145. doi: 10.3390/nu16010145 (PMC10780616; doi:10.3390/nu16010145)
Supplement: Supplementary file 1 [file nutrients-16-00145-s001.zip › FigureS1.pdf]

Online Supplementary Material

Effect of nicotinamide mononucleotide concentration in human milk on neurodevelopmental outcome: the Tohoku Medical Megabank Project Birth and Three-Generation Cohort Study.

Yoshie Saito et al.

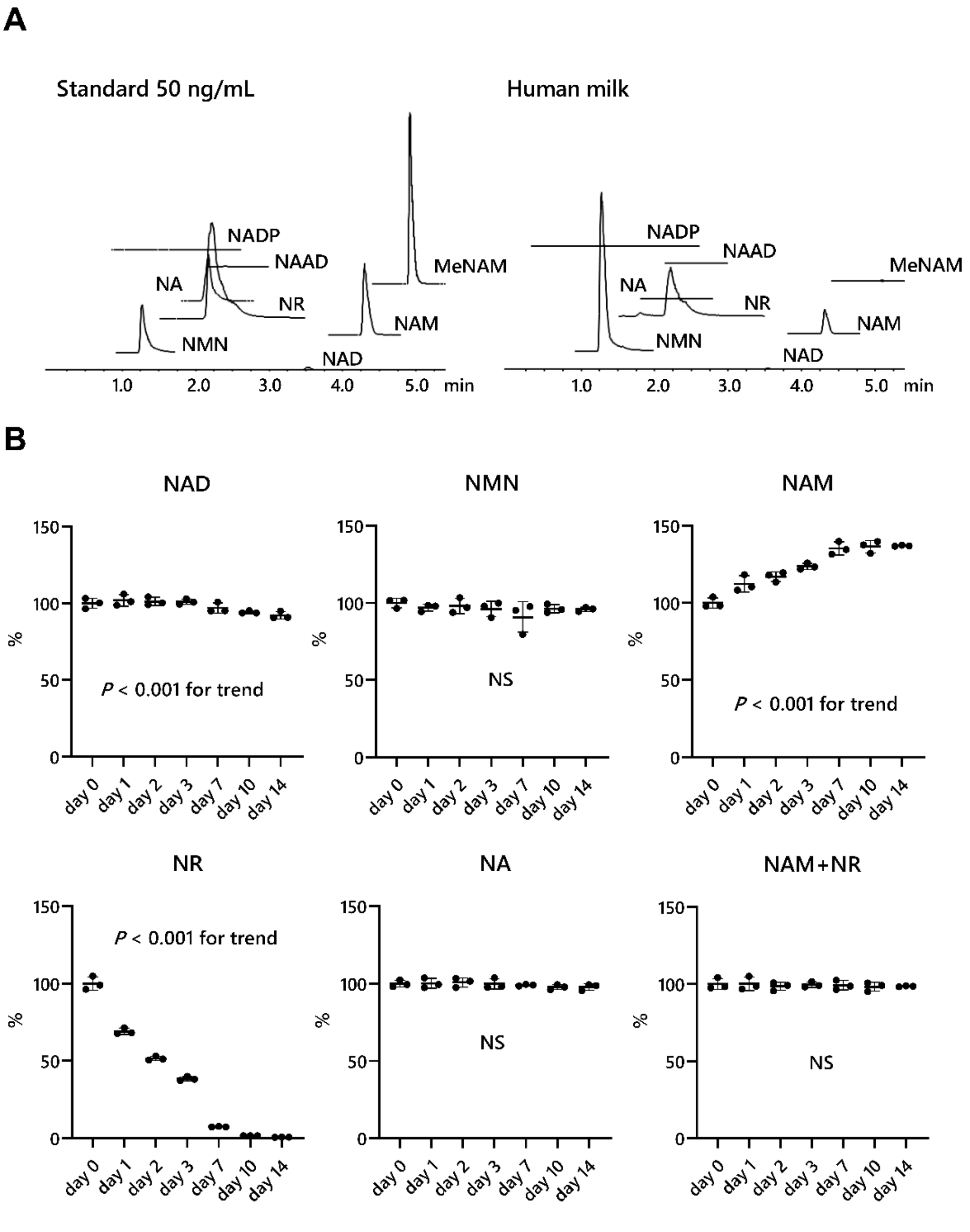

Supplementary Figure S1. A) Analysis of NAD-related substances in breast milk samples. HPLC–MS/MS chromatograms obtained from analysis of standard and human milk for NAD-related substances are shown. Representative chromatograms for a standard solution (50 ng/mL) of the indicated compound are shown on the left,

and those for breast milk samples are shown on the right. B) Measurement of the stability of NAD-related substances. The concentrations of NAD-related substances in breast milk after storage at 4 °C for 14 days were analyzed using HPLC–MS/MS. The percentage of compound remaining after the storage period is shown. The average value on day 0 was set as 100%. Average values are shown (Mean ± SD, *N* = 3; Jonckheere-Terpstra Test). NAD: nicotinamide adenine dinucleotide, NMN: nicotinamide mononucleotide, NAM: nicotinamide, NR: nicotinamide riboside, NA: nicotinic acid, MeNAM: methylnicotinamide, NAAD: nicotinic acid adenine dinucleotide, and NADP: nicotinamide adenine dinucleotide phosphate.
