# Supplementary material for: Effect of Nicotinamide Mononucleotide Concentration in Human Milk on Neurodevelopmental Outcome: The Tohoku Medical Megabank Project Birth and Three-Generation Cohort Study
Source: Nutrients. 2023 Dec 31;16(1):145. doi: 10.3390/nu16010145 (PMC10780616; doi:10.3390/nu16010145)
Supplement: Supplementary file 1 [file nutrients-16-00145-s001.zip › FigureS2.pdf]

Online Supplementary Material

Effect of nicotinamide mononucleotide concentration in human milk on neurodevelopmental outcome: the Tohoku Medical Megabank Project Birth and Three-Generation Cohort Study.

Yoshie Saito et al.

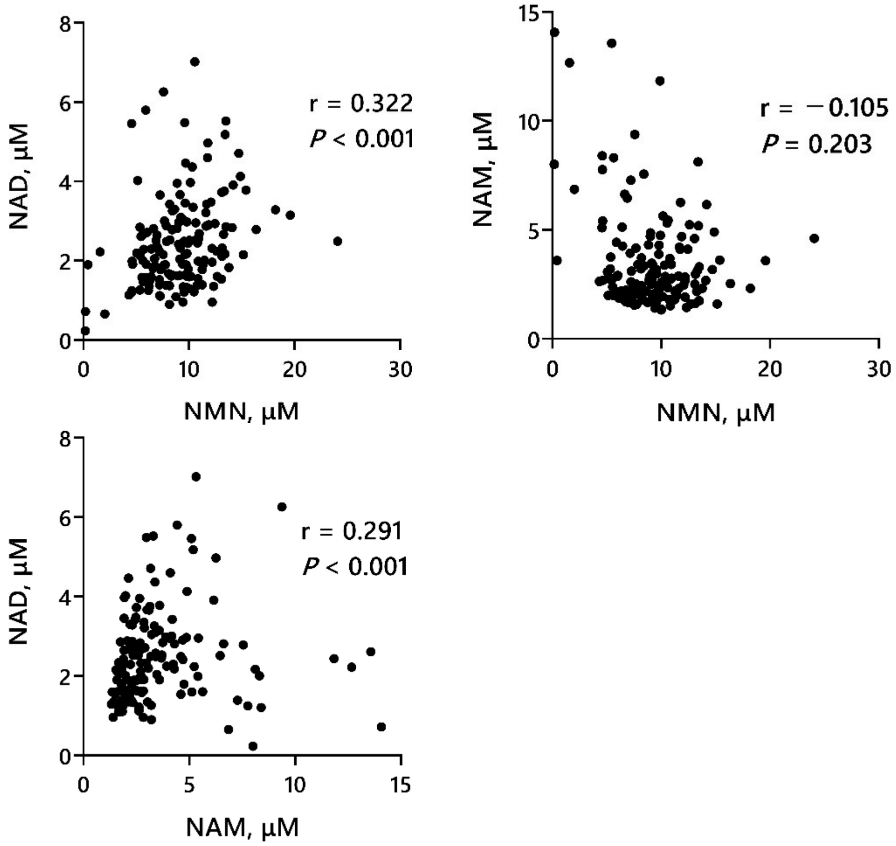

Supplementary Figure S2. Correlation of NAD-related compounds in human milk (Speaman's correlation test). NAD: Nicotinamide adenine dinucleotide, NMN: Nicotinamide mononucleotide, and NAM: Nicotinamide.
