# Supplementary material for: Effect of Nicotinamide Mononucleotide Concentration in Human Milk on Neurodevelopmental Outcome: The Tohoku Medical Megabank Project Birth and Three-Generation Cohort Study
Source: Nutrients. 2023 Dec 31;16(1):145. doi: 10.3390/nu16010145 (PMC10780616; doi:10.3390/nu16010145)
Supplement: Supplementary file 1 [file nutrients-16-00145-s001.zip › TableS1.pdf]

Online Supplementary Material

Effect of nicotinamide mononucleotide concentration in human milk on neurodevelopmental outcome: the Tohoku Medical Megabank Project Birth and Three-Generation Cohort Study.

Yoshie Saito et al.

Supplementary Table S1. Parameters for HPLC–MS/MS analysis of NAD-related compounds.

Analyte		Molecular weight of free form (Da)	Parent ion (m/z)	Daughter ion (m/z)	Cone (v)	Collision (eV)
NAD	Nicotinamide adenine dinucleotide	663.4	664.2	428.1	35	26
NAD_IS	Nicotinamide adenine dinucleotide-$^{13}C^{5}$	668.4	669.1	428.0	6	26
NMN	Nicotinamide mononucleotide	334.2	335.2	123.0	18	12
NMN_IS	Nicotinamide-d4 mononucleotide	338.2	339.1	84.1	20	49
NAM	Nicotinamide	122.1	123.2	79.9	46	14
NAM_IS	Nicotinamide-$^{13}C^{6}$	128.1	129.0	85.4	32	20
NR	Nicotinamide riboside	255.3	255.1	123.1	10	10
NA	Nicotinic acid	123.1	124.0	80.2	16	18
NA_IS	Nicotinic acid-$^{13}C^{6}$	129.1	130.1	83.0	22	24
MeNAM	N-Methylnicotinamide	136.2	137.1	79.9	34	20
NAMN	Nicotinic acid mononucleotide	335.2	336.2	124.0	4	12
NAAD	Nicotinic Acid Adenine Dinucleotide	665.4	665.2	428.2	46	28
NADP	Nicotinamide adenine dinucleotide phosphate	743.4	744.0	604.0	54	18
