# Supplementary material for: Effect of Nicotinamide Mononucleotide Concentration in Human Milk on Neurodevelopmental Outcome: The Tohoku Medical Megabank Project Birth and Three-Generation Cohort Study
Source: Nutrients. 2023 Dec 31;16(1):145. doi: 10.3390/nu16010145 (PMC10780616; doi:10.3390/nu16010145)
Supplement: Supplementary file 1 [file nutrients-16-00145-s001.zip › TableS2.pdf]

Online Supplementary Material

Effect of nicotinamide mononucleotide concentration in human milk on neurodevelopmental outcome: the Tohoku Medical Megabank Project Birth and Three-Generation Cohort Study.

Yoshie Saito et al.

Supplementary Table S2. Matrix-matched calibration range, recovery, ion suppression, and precision of NAD-related compounds.

Analyte	Fitted calibration	Applied weighting	Selected internal standard	Calibrated range in human milk (μM)	Recovery (%)	Ion suppression (%)	Inter-day precision (%)
NAD	Linear	1/x	NAD_IS	1.5–150.7	105.0%	−6.0%	6.4%
NMN	Linear	1/x	NMN_IS	0.6–59.8	91.6%	56.4%	1.4%
NAM	Linear	1/x	NAM_IS	0.3–163.8	89.7%	2.0%	1.0%
NR	Linear	1/x	NAM_IS	0.2–78.4	101.8%	−0.4%	12.2%
NA	Linear	1/x	NA_IS	0.3–162.5	100.5%	−8.0%	8.0%
MeNAM	Linear	1/x	NAM_IS	0.3–146.9	101.6%	−12.2%	-
NAAD	Linear	1/x	NAD_IS	0.3–30.1	107.7%	−17.1%	-
NADP	Linear	1/x	NAD_IS	2.7–26.9	77.7%	10.1%	-

NAD: Nicotinamide adenine dinucleotide, NMN: Nicotinamide mononucleotide, NAM: nicotinamide, NR: Nicotinamide riboside, NA: Nicotinic acid, MeNAM: Methylnicotinamide, NAAD: Nicotinic acid adenine dinucleotide, and NADP: Nicotinamide adenine dinucleotide phosphate.
