# Supplementary material for: Effect of Nicotinamide Mononucleotide Concentration in Human Milk on Neurodevelopmental Outcome: The Tohoku Medical Megabank Project Birth and Three-Generation Cohort Study
Source: Nutrients. 2023 Dec 31;16(1):145. doi: 10.3390/nu16010145 (PMC10780616; doi:10.3390/nu16010145)
Supplement: Supplementary file 1 [file nutrients-16-00145-s001.zip › TableS3.pdf]

# Online Supplementary Material

**Effect of nicotinamide mononucleotide concentration in human milk on neurodevelopmental outcome: the Tohoku Medical Megabank Project Birth and Three-Generation Cohort Study.**

**Yoshie Saito et al.**

**Supplementary Table S3.** Quantiles of NAD-related substances in breast milk.

| Analyte | unit | *N* | mean | SD | Median | min | max | 10% | 25% | 50% | 75% | 90% |
|---|---|---|---|---|---|---|---|---|---|---|---|---|
| NAD | µM | 150 | 2.5 | 1.2 | 2.2 | 0.2 | 7.0 | 1.3 | 1.6 | 2.2 | 3.0 | 4.0 |
| NMN | µM | 150 | 9.2 | 3.5 | 9.2 | 0.2 | 24.1 | 5.4 | 7.0 | 9.2 | 11.2 | 13.3 |
| NAM | µM | 150 | 3.5 | 2.3 | 2.7 | 1.3 | 14.1 | 1.8 | 2.1 | 2.7 | 4.2 | 6.3 |
| NR | µM | 150 | 1.1 | 1.2 | 0.8 | 0.0 | 6.0 | 0.0 | 0.2 | 0.8 | 1.7 | 2.6 |
| NA | µM | 150 | 0.0 | 0.1 | 0.0 | 0.0 | 0.9 | 0.0 | 0.0 | 0.0 | 0.0 | 0.0 |

NAD: Nicotinamide adenine dinucleotide, NMN: Nicotinamide mononucleotide, NAM: Nicotinamide, NR: Nicotinamide riboside, and NA: Nicotinic acid.
