# Supplementary material for: Effect of Nicotinamide Mononucleotide Concentration in Human Milk on Neurodevelopmental Outcome: The Tohoku Medical Megabank Project Birth and Three-Generation Cohort Study
Source: Nutrients. 2023 Dec 31;16(1):145. doi: 10.3390/nu16010145 (PMC10780616; doi:10.3390/nu16010145)
Supplement: Supplementary file 1 [file nutrients-16-00145-s001.zip › TableS4.pdf]

Online Supplementary Material

Effect of nicotinamide mononucleotide concentration in human milk on neurodevelopmental outcome: the Tohoku Medical Megabank Project Birth and Three-Generation Cohort Study.

Yoshie Saito et al.

Supplementary Table S4. Association between NAD-related substances in breast milk and developmental indicators determined via ordinal logistic regression. Analysis of mother–infant pairs excluding infants with a gestational age of less than 37 weeks.

24-month ASQ-3	Crude			Adjusted		
	cOR (95% CI)	Q value		cOR (95% CI)	Q value	
NMN						
Communication	1.12 (1.02, 1.24)	0.021	*	1.15 (1.03, 1.28)	0.014	*
Gross motor	1.12 (1.01, 1.23)	0.024	*	1.15 (1.03, 1.29)	0.014	*
Fine motor	1.12 (1.03, 1.23)	0.014	*	1.14 (1.04, 1.25)	0.011	*
Problem solving	1.21 (1.09, 1.33)	0.001	*	1.19 (1.08, 1.32)	0.002	*
Personal-social	1.14 (1.04, 1.25)	0.014	*	1.20 (1.08, 1.33)	0.002	*

Crude model: (N = 148)
Adjusted model: adjusted for gestational week, household income, alcohol consumption during pregnancy, and number of children (N = 142).
* Q < 0.05; NMN: Nicotinamide mononucleotide; ASQ: Ages and Stages Questionnaire.
