# Supplementary material for: Effect of Nicotinamide Mononucleotide Concentration in Human Milk on Neurodevelopmental Outcome: The Tohoku Medical Megabank Project Birth and Three-Generation Cohort Study
Source: Nutrients. 2023 Dec 31;16(1):145. doi: 10.3390/nu16010145 (PMC10780616; doi:10.3390/nu16010145)
Supplement: Supplementary file 1 [file nutrients-16-00145-s001.zip › TableS5.pdf]

Online Supplementary Material

Effect of nicotinamide mononucleotide concentration in human milk on neurodevelopmental outcome: the Tohoku Medical Megabank Project Birth and Three-Generation Cohort Study.

Yoshie Saito et al.

Supplementary Table S5. Association between NAD-related substances in breast milk and developmental indicators determined using ordinal logistic regression. Analysis of mother–infant pairs excluding infants with low birth weight (< 2,500 g).

24-month ASQ-3	Crude			Adjusted		
	cOR (95% CI)	Q value		cOR (95% CI)	Q value	
NMN						
Communication	1.13 (1.02, 1.25)	0.024	*	1.15 (1.03, 1.29)	0.017	*
Gross motor	1.11 (1.01, 1.23)	0.038	*	1.16 (1.03, 1.30)	0.017	*
Fine motor	1.10 (1.01, 1.20)	0.036	*	1.12 (1.02, 1.23)	0.020	*
Problem solving	1.21 (1.09, 1.33)	0.001	*	1.19 (1.07, 1.32)	0.003	*
Personal-social	1.14 (1.04, 1.26)	0.014	*	1.21 (1.09, 1.35)	0.002	*

Crude model: (N = 134)
Adjusted model: adjusted for gestational week, household income, alcohol consumption during pregnancy, and number of children (N = 129).
* Q < 0.05; NMN: Nicotinamide mononucleotide; ASQ: Ages and Stages Questionnaire.
